# Supplementary material for: Splice-Junction-Based Mapping of Alternative Isoforms in the Human Proteome
Source: Cell Rep. Author manuscript; Available in PMC 2020 Jan 15. (PMC6961840; doi:10.1016/j.celrep.2019.11.026)
Supplement: 3 [file NIHMS1546469-supplement-3.zip › DF2/PXD009737/TestisTrypsin-535-P24043-NLRPEVNLK.pdf]

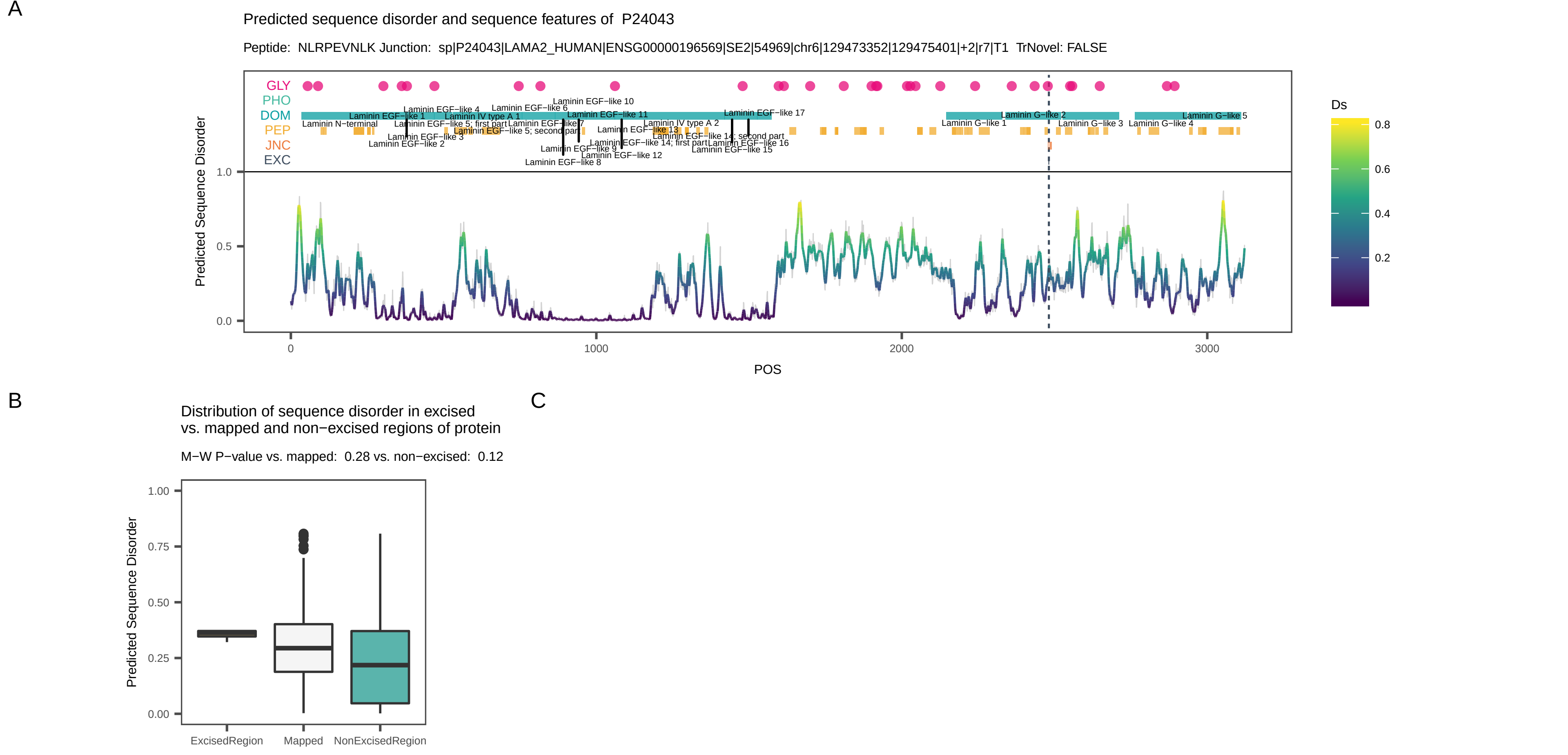

A
Predicted sequence disorder and sequence features of P24043
Peptide: NLRPEVNLK Junction: sp|P24043|LAMA2_HUMAN|ENSG00000196569|SE2|54969|chr6|129473352|129475401|+2|r7|T1 TrNovel: FALSE
GLY
PHO
DOM
PEP
JNC
EXC
Predicted Sequence Disorder
POS
Ds
B
Distribution of sequence disorder in excised vs. mapped and non−excised regions of protein
M−W P−value vs. mapped: 0.28 vs. non−excised: 0.12
Predicted Sequence Disorder
ExcisedRegion
Mapped
NonExcisedRegion
C
